# A Critical Narrative Review of Coaxial Double-Pigtail Stenting Within the LAMS in the Management of Pancreatic Fluid Collections

**DOI:** 10.3390/medicina61081500

**Published:** 2025-08-21

**Authors:** Giuseppe Infantino, Gabriele Rancatore, Dario Quintini, Lucio Carrozza, Dario Ligresti, Marco Giacchetto, Nicoletta Belluardo, Giuseppe Rizzo, Elio D’Amore, Giacomo Emanuele Maria Rizzo, Ilaria Tarantino

**Affiliations:** 1 Gastroenterology and Endoscopy Unit, IRCCS ISMETT (Istituto Mediterraneo per i Trapianti e Terapie ad Alta Specializzazione), University of Pittsburgh Medical Center Italy (UPMCI), CAP 90127 Palermo, Italy; giuseppeinfantino7@gmail.com (G.I.); grancatore@ismett.edu (G.R.); dquintini@ismett.edu (D.Q.); lucio.carrozza@gmail.com (L.C.); dligresti@ismett.edu (D.L.); cmgiacchetto@ismett.edu (M.G.); nbelluardo@ismett.edu (N.B.); girizzo@ismett.edu (G.R.); elio.damore@gmail.com (E.D.); itarantino@ismett.edu (I.T.); 2 Gastroenterology and Endoscopy Unit, Papardo Hospital, CAP 98100 Messina, Italy

**Keywords:** pancreatic fluid collections, LAMS, walled-off necrosis, pancreatic pseudocysts, lumen-apposing metal stents, coaxial double-pigtail plastic stent, coaxial DPPS, PFCs, WON

## Abstract

Endoscopic ultrasound (EUS)-guided drainage using lumen-apposing metal stents (LAMSs) has become the standard for managing pancreatic fluid collections (PFCs), especially walled-off necrosis (WON). However, LAMS-specific adverse events (AEs), including bleeding, stent occlusion, and infection, remain a concern. To mitigate these complications, some experts advocate placing coaxial double-pigtail plastic stents (DPPSs) within LAMSs. This narrative review critically examines the evidence from retrospective and prospective studies, one RCT, and recent meta-analyses on this combined approach. While the routine use of coaxial double-pigtail plastic stents (DPPSs) within LAMSs is not universally supported, emerging data suggest benefits in select high-risk scenarios, such as large WON, debris-rich cavities, or disconnected pancreatic duct syndrome (DPDS), in which coaxial DPPS within LAMSs can reduce occlusion, infection, and recurrence. In addition, the type of LAMS appears to influence safety outcomes: the SPAXUS stent shows lower bleeding and migration rates than the Hot AXIOS. We propose a pragmatic algorithm for the patient-tailored use of coaxial DPPS and discuss technical innovations to improve outcomes. While evidence is still evolving, personalized strategies and future high-quality studies are needed to define the optimal role of coaxial DPPS within LAMSs in the EUS-guided drainage of PFCs.

## 1. Introduction

Pancreatic fluid collections (PFCs), meaning pancreatic pseudocysts (PPs) and walled-off necrosis (WON), are well-recognized complications of acute and chronic pancreatitis [1]. In recent years, endoscopic ultrasound (EUS)-guided drainage has emerged as the first-line approach for managing symptomatic or infected PFCs, offering a minimally invasive alternative to surgical or percutaneous methods [2]. The advent of lumen-apposing metal stents (LAMSs) has further revolutionized endoscopic drainage, enabling efficient internal drainage and direct endoscopic necrosectomy [3,4,5]. LAMSs are self-expanding metal stents with biflanged ends, designed to create a stable fistulous tract between the gastrointestinal lumen and the collection cavity. Their large internal diameter (up to 20 mm) facilitates drainage and instrumentation [6], but has also been associated with specific adverse events (AEs), such as stent occlusion, bleeding, migration, mucosal injury, and buried stent syndrome, particularly in complex necrotic collections or prolonged stent indwelling [7,8]. To mitigate these risks, some endoscopists have adopted the strategy of inserting one or two coaxial double-pigtail plastic stents (DPPSs) within the LAMS. Unlike LAMSs, DPPSs are thin, flexible plastic stents (typically 7–10 Fr) with curled ends aimed at stabilizing the tract, and reducing migration [9]. The rationale for this combined approach is based on several theoretical mechanisms: DPPSs may help prevent occlusion by debris or food impaction, reduce trauma by limiting contact between the metal flanges and the gastrointestinal wall, provide additional anchorage to prevent migration, and offer a prolonged internal drainage route [10,11]. While these potential benefits are biologically plausible, the current clinical impact is still unclear [12]. This narrative review critically evaluates the available evidence on the combined use of LAMSs and coaxial DPPSs within the LAMS in the treatment of PFCs, focusing on current practices, key clinical studies, and areas of ongoing debate.

## 2. Materials and Methods

This study is a narrative review aimed at critically evaluating the clinical evidence surrounding the use of coaxial DPPS within the LAMS for the endoscopic management of PFCs. A comprehensive search of the literature was conducted up to May 2025 in the major databases (Embase, Medline via PubMed, Scopus, and the Cochrane Library). Search terms included keywords such as “pancreatic fluid collections,” “walled-off necrosis,” “pancreatic pseudocysts,” “lumen-apposing metal stents,” and “coaxial double-pigtail plastic stent.” Relevant studies were selected based on the following inclusion criteria: (1) studies including patients with PFCs needing endoscopic drainage (WON or PPs); (2) studies evaluating endoscopic treatments as EUS-guided drainage through the LAMS alone or coaxial DPPS within the LAMS; (3) both single arm and comparative studies. Exclusion criteria were (1) studies in which outcomes were not evaluated; (2) studies including pediatric patients (less than 16 years old); (3) studies published in a language other than English. No restrictions in type of publication (abstract form or full publication) and study design were considered, so both retrospective and prospective studies were screened, including randomized controlled trials and meta-analyses. Technical innovations and consensus recommendations were also reviewed. No formal risk of bias assessment was conducted, as this was not a systematic review. References were curated based on relevance and methodological quality, with emphasis placed on clinical endpoints such as technical and clinical success, AE rates, and recurrence.

## 3. Results

### 3.1. Clinical Data Comparing LAMSs vs. Coaxial DPPS Within LAMSs

**Figure 1:**
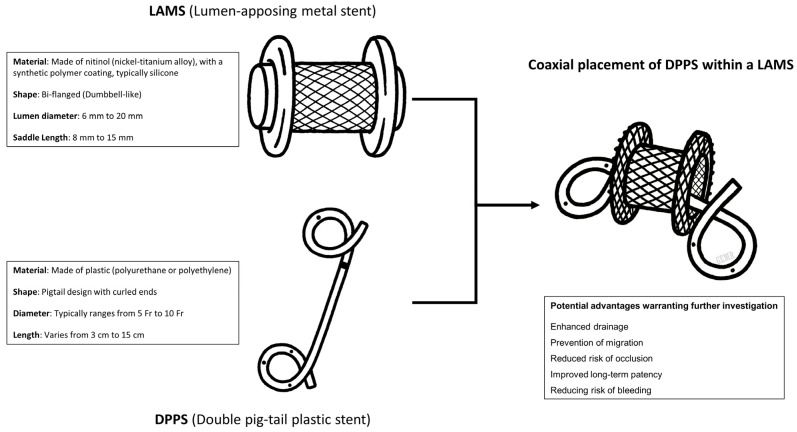
Technical findings of lumen-apposing metal stent (LAMS) and double pigtail plastic stent (DPPS), with potential advantages of placing a coaxial DPPS within the LAMS needing a deeper investigation.

Several studies have evaluated the role of coaxial DPPS within LAMSs for the endoscopic drainage of PFCs, particularly WON and PPs. The technical and structural differences between LAMSs and DPPSs may explain how they could affect clinical outcomes, and their combined use in a coaxial DPPS within the LAMS configuration may offer potential advantages (Figure 1), which this review aimed to explore and analyze in more depth.

However, while the theoretical rationale is strong, clinical evidence has yielded conflicting results, with both supportive and neutral findings reported across retrospective and prospective studies. One of the first studies to examine this technique was a single-center retrospective cohort by Puga et al., which included 41 patients undergoing EUS-guided drainage of PFCs using a LAMS alone (*n* = 21) or coaxial DPPS within the LAMS (*n* = 20). Technical and clinical success rates were similar between groups (85.7 vs. 90%, *p* > 0.99), but the incidence of AEs was significantly higher in the LAMS-alone group (42.9%) compared to the coaxial DPPS within the LAMS group (10.0%) (*p* = 0.04). Bleeding was the most common complication, suggesting a potential protective role of coaxial DPPS within the LAMS in reducing mucosal injury or friction-related trauma from the metal flanges [13]. Comparable findings were reported by Aburajab et al. in a study focused exclusively on PPs (47 patients: 24 LAMS-alone and 23 coaxial DPPS within the LAMS). Clinical success was high in both groups (91% and 100%, respectively, *p* = 0.24). However, infection occurred in 17% of patients treated with a LAMS alone and in none of the coaxial DPPS within the LAMS group (*p* = 0.054). These findings suggested a possible role for coaxial DPPS within the LAMS in preventing the food-related contamination of cystic cavities, particularly when in close proximity to the gastric or duodenal lumen [14]. Both studies suggest that coaxial DPPS within the LAMS might act as a mechanical barrier, potentially improving safety during prolonged indwelling of LAMSs. A more recent retrospective study presented in abstract form further supported the benefit of coaxial DPPS within the LAMS. Among 101 patients undergoing EUS-guided drainage of PFCs, 80 received coaxial DPPSs within the LAMS and 21 received LAMSs alone. The overall AEs rate was significantly lower in the coaxial DPPS within the LAMS group (17.5%) compared to the LAMS-only group (47.6%; *p* = 0.01). Bleeding occurred in 5% of the coaxial DPPS within the LAMS group vs. 28.6% in the LAMS-alone group (*p* = 0.01); perforation in 1.3% vs. 9.5% (*p* = 0.04); and infection in 11.3% vs. 28.6% (*p* = 0.04). Despite being limited to an abstract format, these findings could provide additional support for a protective role of coaxial DPPS within the LAMS in reducing LAMS-related complications, especially if they become available as full publications [15]. In contrast, larger retrospective series have raised doubts regarding the routine use of coaxial DPPS within the LAMS. AbiMansour et al. conducted a large retrospective study involving 185 patients (83 LAMS-alone and 102 coaxial DPPS within the LAMS), including about 75% of WON. Clinical success rates were comparable (75.9% vs. 69.6%, *p* = 0.34), and overall AEs occurred in 15.7% of patients in both groups (*p* = 0.825). Notably, bleeding was more frequent in the coaxial DPPS within the LAMS group (8.8%) than in the LAMS-alone group (2.4%), though this difference was not statistically significant (*p*= 0.067). The authors concluded that routine coaxial DPPS within the LAMS placement did not improve efficacy or safety, though it may still be useful in select subgroups, such as those with disconnected pancreatic duct syndrome (DPDS) or complex WON requiring prolonged drainage. In such situations, indeed, the DPPSs may help maintain long-term tract patency and, potentially, reduce recurrence after LAMS removal. The study underlines the importance of individualized decision-making, and suggests that future prospective research should focus on identifying which subgroups of patients might derive benefit from this adjunctive technique [16,17]. Similar findings were reported by Ali et al. in a single-center retrospective study of 57 patients (21 LAMS-alone and 36 coaxial DPPS within the LAMS). Though clinical success appeared numerically higher in the LAMS-alone group (71.4%) compared to the coaxial DPPS within the LAMS group (58.3%), the difference was not statistically significant (*p* = 0.32). Likewise, AEs occurred in 28.6% and 38.9% of patients, respectively (*p* = 0.43). The most frequent complication was stent obstruction, followed by bleeding, and migration. Based on these findings, the authors concluded that coaxial DPPS within the LAMS did not offer a significant improvement in safety or efficacy, and therefore did not recommend its routine use [18]. A similar lack of benefit was observed in a multicenter retrospective study by Shamah et al., which included 68 patients with various types of PFCs (65% pseudocysts, 25% WON, and 10% postsurgical fluid collections). Patients were divided into two groups: coaxial DPPS within the LAMS (*n* = 35) and LAMS-alone (*n* = 33). Overall clinical success was 94%, with a non-significant trend favoring LAMS-alone (96% vs. 83%). AEs rates were similar (30% vs. 26%, *p* = 0.75), including bleeding, occlusion, perforation, and migration. Subgroup analysis revealed no benefit of coaxial DPPS within the LAMS in any PFC subtype [19].

Other studies have tried to refine patient selection for the placement of coaxial DPPSs within the LAMS. Rossi et al. retrospectively evaluated 49 patients treated with Hot AXIOS LAMSs, 17 of whom received an additional coaxial DPPS within the LAMS. The overall clinical success rate was 93.9%, with AEs occurring in 16.3% of patients. Bleeding occurred in 10.2% of cases (including two deaths). Bleeding was numerically lower in the coaxial DPPS within the LAMS group (5.9%) compared to the LAMS-only group (15.6%), though not statistically significant (*p* = 0.65). The authors concluded that while LAMS drainage is effective, bleeding remains a serious concern, and coaxial DPPSs within the LAMS might offer some protection, warranting further investigation [20]. In a bi-center retrospective study by Haddad et al., 68 patients were analyzed (45 LAMS-alone and 23 coaxial DPPS within the LAMS) using propensity-score matching. Clinical success was 84.4% for LAMS-alone and 95.7% for coaxial DPPS within the LAMS (*p* = 0.18), while AEs occurred in 28.9% vs. 17.4%, respectively (*p* = 0.27). No statistically significant differences were observed for stent occlusion, bleeding, infection, or migration. The authors concluded that the use of coaxial DPPS within the LAMS did not yield a significant clinical advantage [21]. On the other hand, more promising results have emerged from recent studies specifically evaluating WON. In a retrospective study by Perez Estrada et al., subgroup analysis of WON patients revealed that the placement of coaxial DPPSs within the LAMS was associated with significantly fewer endoscopic revisions (1.38 ± 1.50 vs. 4.39 ± 3.97; *p* = 0.042) and earlier LAMS removal (45.75 ± 26.46 vs. 149.00 ± 34.72 days; *p* < 0.001), though overall AEs rates did not differ significantly between groups [22]. These findings were also supported by the only available prospective randomized controlled trial (RCT), conducted by Vanek et al., involving 67 patients with WON randomized to LAMS alone (*n* = 33) or coaxial DPPS within the LAMS (*n* = 34). The coaxial DPPS within the LAMS group showed significantly fewer AEs overall (20.7% vs. 51.5%; *p* = 0.008), particularly in terms of stent occlusion (14.7% vs. 36.3%; *p* = 0.042). Mortality (2.9% vs. 12.1%) and treatment failure (29.4% vs. 48.5%) were also numerically lower in the coaxial DPPS within the LAMS group, though these differences did not reach statistical significance (*p* = 0.197; *p* = 0.109, respectively). Clinical success at 3 months was similar in both groups (91.2% vs. 81.8%; *p* = 0.305). These results support the use of coaxial DPPS within the LAMS in patients with WON, particularly those at high risk of stent-related complications such as occlusion [23]. A summary of studies comparing LAMSs with and without coaxial DPPS for the management of PFCs is presented in Table 1.

### 3.2. Summary of Evidence

The question of whether coaxial DPPSs provide a clinical benefit when placed through LAMSs for EUS-guided drainage of PFCs remains controversial. Several meta-analyses and expert statements have explored this topic (Table 2), producing conflicting conclusions.

Beran et al. [24] conducted one of the earliest meta-analyses directly comparing LAMS alone to coaxial DPPS within the LAMS. Based on five observational studies including 281 patients, the analysis revealed no statistically significant difference in either technical (RR = 1.01, *p* = 0.70) or clinical success (RR = 1.01, *p* = 0.85) between the two groups. Though overall AEs, stent occlusion, and infection rates were numerically lower in the coaxial DPPS within the LAMS group, none of these outcomes reached statistical significance (RR 0.64, *p* = 0.22; RR 0.63, *p* = 0.29; RR = 0.50, *p* = 0.25). The authors emphasized the theoretical appeal of the placement of coaxial DPPSs within the LAMS for reducing complications such as bleeding or obstruction, but ultimately concluded that the existing data do not support a definitive clinical advantage. Limitations of the analysis included small sample sizes, the absence of randomized studies, and inconsistent reporting across studies. Therefore, the authors highlighted the need for high-quality RCTs, particularly focusing on patients with WON, to better assess the true value of this adjunctive technique [24]. Expanding on this, a more recent meta-analysis by Giri et al. pooled data from eight studies, including one RCT, for a total of 454 patients. Again, no difference in clinical success was observed between the LAMS-alone and coaxial DPPS within the LAMS groups (RR 1.00; *p* = 0.98). Additionally, the incidence of AEs was lower in the coaxial DPPS within the LAMS group (RR 1.60, 95% CI 0.95–2.68; *p* = 0.08), suggesting a favorable but non-significant trend toward the use of coaxial DPPSs within the LAMS. Subgroup analyses by complication type (bleeding, infection, and occlusion) also failed to reveal a consistent advantage. The authors concluded that though the routine placement of coaxial DPPSs within the LAMS is not currently supported by evidence, it may be reasonable in select high-risk scenarios, such as large WON with extensive debris where the likelihood of stent occlusion and secondary infection is higher. They also underscored limitations such as heterogeneity in study design and insufficient outcome stratification, reinforcing the call for prospective studies to clarify which patient subgroups may benefit most [25]. This position is broadly reflected in the I-EUS consensus, which addressed the role of coaxial DPPS within the LAMS during PFCs drainage. Though some observational studies report lower bleeding rates with coaxial DPPS within the LAMS, the recommendation remains weak due to the very low certainty of evidence. The panel advised that the decision to place a coaxial DPPS within the LAMS should be individualized, particularly given that recent meta-analyses failed to demonstrate significant improvements in either clinical success or safety outcomes [29,30]. In contrast to Giri et al., a meta-analysis recently published by Gopakumar et al. reported more robust evidence favoring the use of coaxial DPPSs within the LAMS. The authors included six studies (348 patients), largely overlapping with those reviewed by Giri, but applied stricter inclusion criteria, limiting inclusion to full-text and peer-reviewed studies with well-defined outcomes. The authors found a statistically significant reduction in overall AEs (OR 2.22, 95% CI 1.37–3.59) mainly due to the reduction in stent occlusion (OR 2.36, 95% CI 1.12–4.98) in favor of coaxial DPPS within the LAMS. As with previous analyses, no differences were noted for bleeding or stent migration, and clinical success rates remained similar between groups. These findings suggest that coaxial DPPS within the LAMS may meaningfully enhance the safety profile of LAMS, primarily by reducing stent occlusion, a leading cause of treatment failure and secondary infection [26]. Though the conclusions of Giri et al. [25] and Gopakumar et al. [26] diverge, their underlying data sets are largely consistent. Both included the same six comparative studies, including the only available RCT [23]. The difference in findings appears to stem from methodological rigor rather than content. Giri et al. included conference abstracts and studies with incomplete outcome reporting, which may have introduced heterogeneity and diluted the analysis. On the other hand, Gopakumar et al. focused on higher-quality, well-reported full-text studies, thereby increasing the reliability of their conclusions. This suggests that stricter methodological standards may be key to capturing the true benefit of the placement of coaxial DPPSs within the LAMS, particularly in high-risk scenarios such as WON. In addition, more recently, two meta-analyses have further reinforced the potential benefit of coaxial DPPS placement. The first, by AbiMansour et al., included nine studies with 709 patients and found that the use of coaxial DPPS within the LAMS was associated with lower rates of stent occlusion (OR 0.53, 95% CI 0.29–0.96; *p* = 0.004) and infection (OR 0.55, 95% CI 0.35–0.84; *p* = 0.001), without significant differences in clinical success, bleeding, or overall AEs. These results support the hypothesis that coaxial DPPS within the LAMS may offer preventive benefits, especially by lowering the risk of occlusion and secondary infection, two key contributors to treatment failure and recurrence. Importantly, this meta-analysis included the largest patient cohort to date, and supports the direction already suggested by prior studies and the only available RCT [27]. The second, more recent meta-analysis by Kamal et al., pooling data from 10 studies and 685 patients, confirmed that the addition of coaxial DPPSs within the LAMS significantly reduced the overall rate of AEs (RR 0.58, 95% CI 0.40–0.87; *p* = 0.007) and infections (RR 0.46, 95% CI 0.24–0.85; *p* = 0.01), with no significant differences in clinical success (RR 1.03, 95% CI 0.94–1.13; *p* = 0.53), stent occlusion (*p* = 0.06), bleeding (*p* = 0.18), or stent migration (*p* = 0.78) [28]. These concordant findings across increasingly robust data sets lend further credibility to the selective use of coaxial DPPS within the LAMS as a means to enhance the safety profile of EUS-guided drainage in appropriate clinical scenarios.

### 3.3. Type of LAMS: Does It Matter?

The type of LAMS may significantly influence the safety profile of EUS-guided drainage for PFCs, particularly for WON. The first widely adopted LAMS was the Hot AXIOS (Boston Scientific, Marlborough, MA 01752-1234, USA), and it has been associated with higher rates of delayed bleeding, ranging from 13.4% to 32.4% in retrospective and post-marketing studies [31,32,33]. This AE is believed to result from mechanical trauma caused by the metal flanges in contact with vascularized cavity walls. To address these concerns, the SPAXUS stent (Taewoong Medical, Gimpo-si 10022, Gyeonggi-do, Republic of Korea), introduced in 2016, features a softer profile, rounded edges, and foldable flanges aimed at reducing strain and minimizing tissue trauma [34,35]. Preliminary data suggest a reduced bleeding risk [36,37]. In a recent multicenter, propensity score-matched study including 264 patients, bleeding requiring intervention was less frequent with SPAXUS compared to Hot AXIOS (1.5% vs. 6.8%; *p* = 0.03), and SPAXUS was independently associated with lower bleeding risk in both univariate (*p* = 0.03) and multivariate (*p* = 0.04) analysis. Overall AEs were also significantly lower in the SPAXUS group (3.0% vs. 9.8%; *p* = 0.04) [38]. These findings were corroborated by a recent systematic review and meta-analysis by Kumar et al., including 37 studies, which offered a comprehensive comparison between these two devices. Though technical and clinical success were comparable for AXIOS (97.7% and 90.9%, respectively) and SPAXUS (98.2% and 93.5%, respectively), the AXIOS group had significantly higher rates of AEs (20.4% vs. 7.6%; *p* < 0.01), bleeding (7.0% vs. 1.8%; *p* < 0.01), and stent migration (4.2% vs. 0.9%; *p* < 0.05). Despite substantial heterogeneity across studies (I^2^ > 60%), the consistent direction of the effect estimates seems to support the hypothesis that the SPAXUS may be associated with a lower incidence of clinically significant complications compared to the AXIOS [39]. These data underscore the importance of considering the stent type in the clinical outcomes of EUS-guided drainage for PFCs when evaluating the use of coaxial DPPSs within the LAMS.

### 3.4. Optimizing LAMS-To-DPPS Transition in High-Risk Patients: Strategies and Innovation

As mentioned before, in patients with DPDS the maintenance of long-term internal drainage is essential to prevent recurrence of PFCs in the event of LAMS placement and subsequent removal. Persistent leakage of pancreatic secretions from the disconnected duct can lead to relapse, even after initial resolution. Several studies have indicated that the placement of a coaxial DPPS after LAMS removal can significantly reduce this risk by maintaining a durable drainage route through the fistula [40,41]. To optimize this transition, particularly in high-risk patients with DPDS or rapidly collapsing cavities, two technical innovations have been developed. The first, described by Mukai et al., is the “*puzzle ring technique*,” which involves inserting a coaxial DPPS within the LAMS through the LAMS 7–10 days before its removal. This interval allows granulation tissue to stabilize the plastic stent within the tract. In a cohort of 18 patients with WON and/or DPDS, the technique achieved a technical success rate of 94.4%, which was significantly higher compared with conventional methods (61.8%, *p* = 0.02), and showed no recurrence during over one year of follow up [17]. The second innovation, presented by Kato et al., involves a specially designed multi-loop, semi-pigtail plastic stent that is placed concurrently with the LAMS and remains in place after LAMS removal, even in collapsed cavities. Its unique shape prevents accidental dislodgement, and ensures continued internal drainage [42]. Together, these strategies shift the clinical question from whether to use a coaxial DPPS within the LAMS to when and how to deploy it most effectively and safely. In this evolving field, such tailored approaches offer the potential to reduce complications and improve outcomes in patients at the highest risk of recurrence.

### 3.5. A Pragmatic Approach to the Selective Use of Coaxial DPPSs

**Figure 2:**
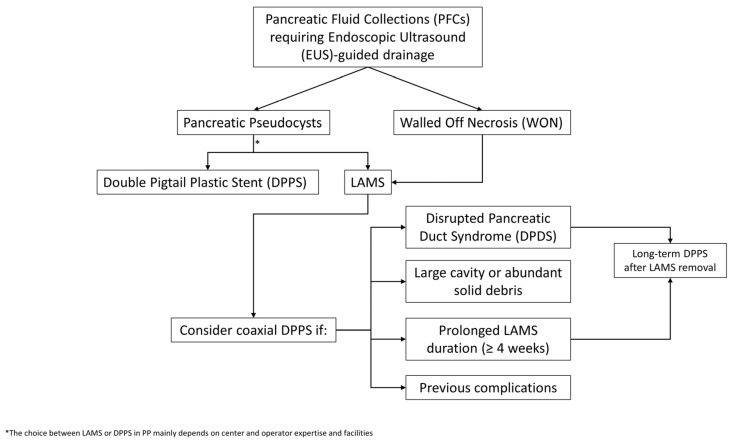
Proposed algorithm for the selective use of a coaxial DPPS within the LAMS during LAMS-assisted EUS-guided drainage of PFCs.

Our review predominantly includes retrospective studies, which are inherently subject to several potential confounders, such as selection bias and heterogeneity in patient populations and variability in procedural expertise across centers. Furthermore, many of the available studies are small and thus underpowered, which may result in non-significant statistical findings despite clinically meaningful trends. These methodological limitations reduce the strength and the generalizability of the reported results and should therefore be carefully considered when interpreting the current evidence. In this context, a pragmatic, patient-tailored algorithm can aid in guiding clinical decision-making regarding coaxial DPPS placement during and after LAMS deployment. The initial step involves distinguishing between different types of collections. In PPs, which carry a low risk of complications, the placement of a LAMS alone is generally sufficient in most cases, even if different factors need to be considered: center and operator expertise and, furthermore, DPPSs have a lower cost, so many centers indistinctly use LAMSs or DPPSs in the context of PPs. In contrast, in patients with WON, the risk of AEs such as stent occlusion, infection, or recurrence is higher. In this regard, the addition of a coaxial DPPS may be considered as a preventive strategy in select high-risk patients. These include those with very large necrotic cavities or abundant solid debris and requiring multiple necrosectomy sessions, patients with prolonged LAMS indwelling time (more than four weeks), those with previously complications such as migration, early occlusion or infection, and cases with suspected or confirmed DPDS. Notably, the presence of DPDS represents a key factor in therapeutic planning because the disruption of the main pancreatic duct leads to continuous leakage of pancreatic secretions, which may favor recurrence of the PFCs after LAMS removal. In this setting, it is essential to ensure long-term internal drainage, and the placement or maintenance of an internal plastic stent represents a crucial measure to prevent relapse. Finally, at the time of LAMS removal, it is essential to avoid the sudden interruption of internal drainage in high-risk patients such as those with DPDS or partially unresolved cavities, so keeping an internal plastic stent may serve as an effective precaution to prolong the therapeutic benefit already achieved. A proposed approach to the endoscopic management of PFCs requiring EUS-guided drainage is shown in Figure 2. Moreover, it is still not time to include the type of LAMSs in an algorithm, despite the encouraging data supporting its use [38,39]. These findings, while promising, still require larger-scale and prospective comparative research to confirm their generalizability and impact across diverse clinical settings. It appears that the benefit of coaxial DPPS placement within LAMSs is not universal, but is most likely to be observed in high-risk clinical scenarios. Low-risk PFCs, such as PPs or WON with a Quadrant-Necrosis-Infection (QNI) score < 2.5, necrosis < 60%, absence of infection, and minimal solid debris, can generally be managed with LAMSs alone [43]. High-risk PFCs could be considered those WON with QNI ≥ 2.5, necrosis ≥ 60%, infected collections, abundant solid debris requiring multiple necrosectomies, prior LAMS-related complications (e.g., early occlusion, migration), or anticipated LAMS dwell time beyond four weeks, and they could prompt the consideration of adding a coaxial DPPS to mitigate the risks of stent occlusion, infection, and recurrence.

Management of LAMS removal should also be individualized. If complete resolution is achieved and no DPDS is present, stent removal without replacement is reasonable. In contrast, if a persistent cavity or DPDS is identified, maintaining long-term internal drainage with a plastic stent may be warranted to prevent recurrence.

## 4. Conclusions

While the routine placement of coaxial DPPSs within the LAMS is conceptually attractive and physio-pathologically plausible, current evidence remains inconclusive. Most available studies are retrospective and heterogeneous, and randomized data are limited. Recent trials and meta-analyses suggest that the benefit of coaxial DPPS within the LAMS should be limited to high-risk scenarios, such as complex WON or suspected DPDS. Our overview allows for a clear and detailed view of the evidence in this field. Notably, the majority of available studies on LAMSs involve the AXIOS stent, while emerging data on alternative devices, such as the SPAXUS stent, suggest considering this variable in future prospective studies as a potential modifier of the efficacy and safety of coaxial DPPS within the LAMS. Emerging technical refinements and better patient stratification will likely help define the optimal strategy for minimizing complications and maximizing clinical outcomes. In this evolving field, clinical judgment remains essential, and a personalized, case-by-case, and multidisciplinary approach is warranted while awaiting more definitive evidence.

## Figures and Tables

**Table 1:** Summary of studies comparing lumen-apposing metal stent (LAMS) with and without coaxial double pigtail plastic stent for the management of pancreatic fluid collections (PFCs).

Study	Year	Study Design	No. of Pts	Type of PFCs	Technique	Clinical Success (%)	Adverse Events (%)	Main Adverse Event	Conclusion
**Puga et al. [13].**	2018	Retrospective. Single-center	41 (21 LAMS; 20 coaxial DPPS within the LAMS)	PP and WON	LAMS ± coaxial- DPPS	LAMS 85.7% Coaxial DPPS within the LAMS 90% *p* > 0.99	42.9% (LAMS) vs. 10% (coaxial DPPS within the LAMS) *p* = 0.04	Bleeding (LAMS 23.8% vs. coaxial DPPS within the LAMS 5%) *p* = 0.18 Infection (LAMS 14.3% vs. coaxial DPPS within the LAMS 5%) *p* = 0.61	Coaxial DPPS within the LAMS associated with fewer complications
**AbiMansour et al. [16].**	2024	Retrospective. Single-center	185 (83 LAMS; 102 coaxial DPPS within the LAMS)	~ 75% WON ~ 25% PP	LAMS ± coaxial-DPPS	15.7% in both groups LAMS: 75.9% Coaxial DPPS within the LAMS: 69.9% *p* = 0.34	15.7 in both groups *p* = 0.825	Bleeding: (LAMS 2.4% vs. coaxial DPPS within the LAMS: 8.8%) *p* = 0.067 Infection (LAMS 7.22% vs. coaxial DPPS within the LAMS 3.92%) *p* = 0.322 LAMS migration (LAMS 3.61% vs. coaxial DPPS within the LAMS 0.98%) *p* = 0.220 LAMS occlusion (LAMS 2.40% vs. coaxial DPPS within the LAMS 2.94%) *p* = 0.825	No benefit with coaxial DPPS within the LAMS; may help in select cases (e.g., DPDS)
**Aburajab et al. [14].**	2018	Retrospective. Single-center	47 (24 LAMS; 23 coaxial DPPS within the LAMS)	PP only	LAMS ± coaxial-DPPS	LAMS: 91% Coaxial DPPS within the LAMS: 100% *p* = 0.24	NR	Infection: (LAMS 17% vs. coaxial DPPS within the LAMS 0%) *p* = 0.054 LAMS migration: (LAMS 4% vs. coaxial DPPS within the LAMS 0%) *p* = 0.50 Perforation: (LAMS 4% vs. coaxial DPPS within the LAMS 0%) *p* = 0.51	Coaxial DPPS within the LAMS may reduce infection risk in PPs by blocking food entryway
**Ali et al. [18].**	2019	Retrospective. Single-center	57 (21 LAMS; 36 coaxial DPPS within the LAMS)	75.4% WON 34.6% PP	LAMS ± coaxial-DPPS	LAMS: 71.4% Coaxial DPPS within the LAMS: 58.3% *p* = 0.32	LAMS: 28.6% Coaxial DPPS within the LAMS: 38.9% *p* = 0.43	Stent obstruction > bleeding/migration Bleeding: (LAMS 16.6% vs. coaxial DPPS within the LAMS 14.2%) LAMS occlusion: (LAMS 66.6% vs. coaxial DPPS within the LAMS 42.8%) LAMS migration (LAMS 16.6% vs. coaxial DPPS within the LAMS 42.8%)	No benefit with coaxial DPPS within the LAMS in terms of efficacy or safety
**Rossi et al. [20].**	2020	Retrospective. Single-center (Congress Abstract)	49 (32 LAMS; 17 coaxial DPPS within the LAMS)	NR	LAMS ± coaxial-DPPS (Hot AXIOS)	93.9% overall	Overall AEs 16.3%	Bleeding: (LAMS 15.6% vs. coaxial DPPS within the LAMS 5.9%) *p* = 0.65	Trend toward less bleeding with coaxial DPPS within the LAMS, though not significant
**Estrada et al. [22].**	2022	Retrospective. Single-center (Abstract)	70 overall	64.2% WON 30% PPs 5.8 post-surgical	LAMS ± coaxial-DPPS	LAMS: 87.9% Coaxial DPPS within the LAMS: 100%	AEs LAMS: 24.1% Coaxial DPPS within the LAMS: 11.1%	Bleeding (LAMS: 3.44% coaxial DPPS within the LAMS: 0%)	No significant difference in the AEs rate between the two groups. Subgroup analysis of patients with WON. Coaxial DPPS within the LAMS was associated with significantly less endoscopic revision (*p* = 0.042) and shorter time to LAMS removal (*p* < 0.001).
**Aujla et al. [15].**	2023	Retrospective. Single- center (Abstract)	101 (21 LAMS; 80 coaxial DPPS within the LAMS)	NR	LAMS ± coaxial-DPPS	NR	Overall AEs: LAMS: 47.6% Coaxial DPPS within the LAMS: 17.5% *p* = 0.01	Bleeding (LAMS: 28.6% Coaxial DPPS within the LAMS: 5%) *p* = 0.01 Perforation (LAMS: 9.5% Coaxial DPPS within the LAMS: 1.3%) *p* = 0.04 Infection (LAMS: 28.6% Coaxial DPPS within the LAMS: 11.3%) *p* = 0.04	The overall AEs rate was significantly lower in the coaxial DPPS within the LAMS group (17.5%) compared to the LAMS-only group (47.6%; *p* = 0.01)
**Haddad et al. [21].**	2023	Retrospective. Bicentric, propensity-matched	68 (45 LAMS; 23 coaxial DPPS within the LAMS)	61.8% WON 30.9% PP 7.3% PSFC	LAMS ± coaxial-DPPS	LAMS: 84.4% Coaxial DPPS within the LAMS: 95.7% *p* = 0.18	LAMS 28.9% Coaxial DPPS within the LAMS 17.4% *p* = 0.27	LAMS occlusion 8.7% vs. 17.8% (*p* = 0.38) Infection 8.7% vs. 8.9% (*p* = 0.97) Bleeding 4.4% vs. 6.7% (*p* = 0.48) LAMS migration 4.4% vs. 8.9% (*p* = 0.45)	No significant difference in outcomes
**Shamah et al. [19].**	2022	Retrospective. Multicenter	68 (35 LAMS; 33 coaxial DPPS within the LAMS)	25% WON 65% PP 10% PSFC	LAMS ± coaxial-DPPS	LAMS: 96% Coaxial DPPS within the LAMS: 83% *p* = 0.67	LAMS 30% Coaxial DPPS within the LAMS 26% *p* = 0.75	Bleeding (LAMS 9% vs. coaxial DPPS within the LAMS 8%) *p* = 0.75 Migration (LAMS 9% vs. coaxial DPPS within the LAMS 15%) *p* = 0.46 Perforation (LAMS 6% vs. coaxial DPPS within the LAMS 2%) *p* = 0.78 Occlusion (LAMS 6% vs. coaxial DPPS within the LAMS 0%) *p* = 0.49	No significant benefit of coaxial DPPS within the LAMS in clinical or safety outcomes
**Vanek et al. [23].**	2023	Prospective. Bicentric RCT	67 (33 LAMS; 34 coaxial DPPS within the LAMS)	WON	LAMS ± coaxial-DPPS	LAMS: 81.8% Coaxial DPPS within the LAMS: 91.2% *p* = 0.305	LAMS 51.5% Coaxial DPPS within the LAMS 20.7% *p* = 0.008	Stent occlusion (14.7% vs. 36.3%. *p* = 0.042) Bleeding (12.1% vs. 5.9%, *p* = 0.427) LAMS migration (6.1% vs. 0%, *p* = 0.239)	Coaxial DPPS within the LAMS significantly reduced global AEs and occlusion

LAMS: lumen-apposing metal stent; DPPS: double pigtail plastic stent; PPs: pancreatic pseudocysts; WON: walled-off necrosis; PSFC: post-surgical fluid collection; AEs: adverse events; NR: not reported.

**Table 2:** Summary of meta-analyses comparing lumen-apposing metal stent with and without coaxial DPPS for the management of pancreatic fluid collections.

Study and Year	No. of Studies	No. of Patients	Technical Success	Clinical Success	Overall AEs	Stent Occlusion	Infection	Perforation	Stent Migration	Bleeding	Conclusion
**Beran et al. [24].**	5	281: overall 137: coaxial DPPS within the LAMS 144: LAMS	RR = 1.01 95% Cl: 0.97–1.04 *p* = 0.70	RR = 1.01 95% Cl: 0.88–1.17 *p* = 0.85	RR = 0.64 95% Cl: 0.32–1.29 Favorable trend towards coaxial DPPS within the LAMS, but not statistically significant, *p* = 0.22	RR = 0.63 95% Cl: 0.27–1.49 Favorable trend towards coaxial DPPS within the LAMS, but not statistically significant, *p* = 0.29	RR = 0.50 95% Cl: 0.15–1.64 Favorable trend towards coaxial DPPS within the LAMS, but not statistically significant, *p* = 0.25	RR = 0.40 95% Cl: 0.06–2.78 Favorable trend towards coaxial DPPS within the LAMS, but not statistically significant, *p* = 0.42	RR = 1.29 95% Cl: 0.50–3.34 *p* = 0.60	RR = 0.65 95% Cl: 0.25–1.74 *p* = 0.39	Coaxial DPPS within the LAMS did not show statistically significant benefits in terms of efficacy or safety
**Giri et al. [25].**	8	454: overall	NR	RR = 1.00 95% Cl: 0.87–1.14 *p* = 0.98	RR = 1.60 95% Cl: 0.95–2.68 *p* = 0.08	RR = 1.72 95% Cl: 0.90–3.27 *p* = 0.10	RR = 1.78 95% Cl: 0.34–9.47 *p* = 0.50	NR	RR = 0.81 95% Cl: 0.33–2.01 *p* = 0.65	RR = 1.80 95% Cl: 0.83–3.88 *p* = 0.14	Coaxial DPPS within the LAMS was not associated with decreased AEs rates or better clinical outcomes
**Gopakumar et al. [26].**	6	348 overall 177: LAMS 171: coaxial DPPS within the LAMS	OR: 0.53 (95% CI 0.15–1.83)	OR: 1.10 (0.598–2.05)	OR: 2.22 (1.37–3.59)	NR	NR	NR	OR: 0.95 (0.40–2.23)	OR: 1.84 (0.77–4.38)	Coaxial DPPS within the LAMS can mitigate the overall adverse events observed with LAMS
**AbiMansour et al. [27]**	9	709 overall 388: LAMS 371: coaxial DPPS within the LAMS	OR: 1.08 (0.59–1.96)	OR: 0.96 (0.48–1.89)	OR: 0.57 (0.25–1.29)	OR: 0.53 (0.29–0.96)	OR: 0.53 (0.29–0.96)	NR	OR:1.03 (0.36–2.90)	OR:0.61 (0.22–1.67)	Coaxial DPPS within the LAMS placement with LAMS for PFCs drainage was associated with a reduced risk of LAMS occlusion and PFCs infection
**Kamal et al. [28].**	10	685 overall	NR	RR = 1.03 95% CI: 0.94–1.13 *p* = 0.53	RR = 0.58 95% CI: 0.40, 0.87 *p* = 0.007	RR = 0.57 95% CI: 0.31–1.03 *p* = 0.06	RR = 0.46 95% CI: 0.24–0.85 *p* = 0.01	NR	RR = 0.89 95% CI: 0.38–2.08 *p* = 0.78	RR = 0.58 95% CI: 0.26–1.30 *p* = 0.18	Addition of coaxial DPPS within the LAMS decreased the risk of AEs (infection) in patients with PFCs

LAMS: lumen-apposing metal stent; DPPS: double pigtail plastic stent; AEs: adverse events; RR: relative risk; OR: odds ratio; CI: confidence interval; NR: not reported.
